# Bioactive metabolites of *Asparagopsis* stabilized in canola oil completely suppress methane emissions in beef cattle fed a feedlot diet

**DOI:** 10.1093/jas/skae109

**Published:** 2024-04-22

**Authors:** Frances C Cowley, Robert D Kinley, Sigrid L Mackenzie, Marina R S Fortes, Chiara Palmieri, Gamaliel Simanungkalit, Amelia K Almeida, Breanna M Roque

**Affiliations:** School of Environmental and Rural Science, University of New England, Armidale, NSW 2351, Australia; FutureFeed Pty Ltd, Townsville, QLD 4811, Australia; School of Environmental and Rural Science, University of New England, Armidale, NSW 2351, Australia; School of Chemistry and Molecular Biosciences, The University of Queensland, St Lucia, QLD 4072, Australia; School of Chemistry and Molecular Biosciences, The University of Queensland, St Lucia, QLD 4072, Australia; School of Environmental and Rural Science, University of New England, Armidale, NSW 2351, Australia; School of Environmental and Rural Science, University of New England, Armidale, NSW 2351, Australia; FutureFeed Pty Ltd, Townsville, QLD 4811, Australia

**Keywords:** Asparagopsis, carbon neutral initiatives, methanogenesis, rumen fermentation, red seaweed, sustainability

## Abstract

*Asparagopsis taxiformis* (***Asparagopsis***) has been shown to be highly efficacious at inhibiting the production of methane (**CH**_**4**_) in ruminants. To date, *Asparagopsis* has been primarily produced as a dietary supplement by freeze-drying to retain the volatile bioactive compound bromoform (**CHBr**_**3**_) in the product. Steeping of *Asparagopsis* bioactive compounds into a vegetable oil carrier (**Asp-Oil**) is an alternative method of stabilizing *Asparagopsis* as a ruminant feed additive. A dose–response experimental design used 3 Asp-Oil-canola oil blends, low, medium, and high Asp-Oil which provided 17, 34, and 51 mg *Asparagopsis* derived CHBr_3_/kg dry matter intake (DMI), respectively (in addition to a zero CHBr_3_ canola oil control), in a tempered-barley based feedlot finisher diet, fed for 59 d to 20 Angus heifers (five replicates per treatment). On four occasions, live weight was measured and CH_4_ emissions were quantified in respiration chambers, and blood, rumen fluid, and fecal samples were collected. At the end of the experiment, all animals were slaughtered, with carcasses graded, and samples of meat and edible offal collected for testing of consumer sensory qualities and residues of CHBr_3_, bromide, and iodide. All Asp-Oil treatments reduced CH_4_ yield (g CH_4_/kg DMI, *P* = 0.008) from control levels, with the low, medium, and high Asp-Oil achieving 64%, 98%, and 99% reduction, respectively. Dissolved hydrogen increased linearly with increasing Asp-Oil inclusion, by more than 17-fold in the high Asp-Oil group (*P* = 0.017). There was no effect of Asp-Oil treatment on rumen temperature, pH, reduction potential, volatile fatty acid and ammonia production, rumen pathology, and histopathology (*P* > 0.10). There were no differences in animal production and carcass parameters (*P* > 0.10). There was no detectable CHBr_3_ in feces or any carcass samples (*P* > 0.10), and iodide and bromide residues in kidneys were at levels unlikely to lead to consumers exceeding recommended maximum intakes. Overall, Asp-Oil was found to be safe for animals and consumers of meat, and effective at reducing CH_4_ emissions and yield by up to 99% within the range of inclusion levels tested.

## Teaser

A feed ingredient containing bioactive metabolites of *Asparagopsis* stabilized in canola oil was able to virtually eliminate methane emissions from grain-fed cattle, without impacting animal health or production, or consumer safety.

## Introduction

The red macroalgae *Asparagopsis taxiformis* (***Asparagopsis***) has been shown to be highly efficacious at inhibiting the production of enteric methane (**CH**_**4**_) in ruminants: suppressing CH_4_ yield (g CH_4_/kg DM intake) by 98% (Kinley et al., 2020) in beef cattle provided supplementary*Asparagopsis* as freeze-dried (**FD**) whole algal biomass **(FD-Asp**). To date, the use of FD processing for *Asparagopsis* is the most protective way to dewater the biomass (Vucko et al., 2017) while retaining the volatile bioactive secondary metabolites (Paul et al., 2006), and of these, bromoform (**CHBr**_**3**_) is the most abundant in the FD-Asp product (Machado et al., 2016). Subsequently, FD-Asp has been consistently effective as an enteric CH_4_ inhibitor when incorporated in feeds and supplements for red meat and dairy production (Roque et al., 2019, 2021; Kinley et al., 2020). Considering the energy-intensive nature of FD and the logistics of immediately flash-freezing large quantities of seaweed at harvest there is considerable interest in alternative processing technologies. The FD process may be logistically challenging to scale-up (Magnusson et al., 2020) and FD-Asp may be variable in sensitivity potentially shortening its shelf life under exposure to harsh conditions (Tan et al., 2022). Subsequently there is incentive for cost-effective alternative stabilizing technologies to provide *Asparagopsis* products suitable for variable feeding systems. The red meat and dairy industries would benefit from more options for delivery of *Asparagopsis* to livestock with fit-for-purpose attributes for the multitude of feeding systems.

Vegetable oils are a common ingredient in total mixed rations (**TMR**) and are sometimes incorporated in supplements for ruminants. The physical properties and energy-dense nature of vegetable oils enhance diet quality and have wide acceptance in the feedlot industry, with typical inclusions of 2% to 6% of diet dry matter (**DM**) intake (**DMI**; Zinn and Jorquera, 2007). With such widespread use and compatibility as a carrier, vegetable oil has evolved as a potentially viable option for delivery of the antimethanogenic components of *Asparagopsis* in the red meat and dairy industries. Magnusson et al. (2020) describe a technique for steeping *Asparagopsis* in edible oil resulting in the secondary metabolites being stabilized in the oil (**Asp-Oil**). Compared to FD-Asp, the resulting Asp-Oil composition has been confirmed to exhibit improved shelf life under harsh conditions (Tan et al., 2022) and is equally antimethanogenic (Kinley et al., 2022).

As with FD-Asp and many other sensitive feed ingredients, the Asp-Oil requires storage care to prevent extended exposure to sunlight, moisture, and high temperatures. That said, Asp-Oil was demonstrated to suffer minimal loss of CHBr_3_ when stored for 24 wk in the dark at temperatures up to 40 °C (Tan et al., 2022). The study highlighted that the key factor to extend shelf life for both FD-Asp and Asp-Oil was to insure a sealed container preferably with limited headspace. Recently a 275-d feedlot demonstration with long-fed Wagyu cattle receiving Asp-Oil in a TMR provided strong evidence of CHBr_3_ content stability (Cowley et al., 2023). The Asp-Oil was stored at ambient temperature, protected from sunlight, and sealed when not in use, in a 1,000 L bulk container. There was no change in CHBr_3_ content of the Asp-Oil for the duration of the more than 9-mo feeding period.

Although stability appears confirmed, the efficacy and dose–response of Asp-Oil in feedlot TMR requires further elucidation. Previous studies with FD-Asp have elucidated a dose–response relationship with no evidence of effect on animal welfare or food product quality and safety allowing for provision of a range of effective inclusion levels (**REIL**) dependent on CHBr_3_ content in the TMR on a mg CHBr_3_/kg DMI basis (Kinley et al., 2020; Roque et al., 2021). This knowledge allows for formulation of a diet including FD-Asp for high grain TMR’s for beef feedlots and prediction of the expected level of CH_4_-inhibition. There is no corresponding knowledge to support equivalent functionality and no REIL exists for Asp-Oil which represents a knowledge gap for this promising format of delivery of *Asparagopsis* derived antimethanogenic secondary metabolites. Otherwise, the widespread use of vegetable oils in feedlot diets suggests that such a product may be readily adopted in grain-fed beef production. However, Asp-Oil must first be demonstrated to be safe (for animals and consumers of the products) and efficacious at inhibiting CH_4_ production.

The effectiveness of Asp-Oil at suppressing enteric CH_4_ emissions was the primary research question and was tested in open-circuit respiration chambers using 20 individually fed Angus heifers. The aim of this study was to demonstrate the antimethanogenic effectiveness and safety of Asp-Oil fed to beef cattle; whether the antimethanogenic capability of Asp-Oil was equivalent to FD-Asp, as demonstrated in published studies; and the response of beef cattle to increasing inclusions of Asp-Oil on a CHBr_3_/kg DMI basis in a feedlot finishing diet. A secondary objective was to measure the impact of these inclusion levels on indicators of cattle health, physiology, and performance, the mass balance of CHBr_3_, quantify residues of *Asparagopsis*, and evaluate effects on eating quality of the meat. It was hypothesized, from previous work testing FD-Asp (Li et al., 2018; Kinley et al., 2020), that CH_4_ emissions would respond in a linear manner over the range of CHBr_3_ inclusion tested (up to 51 mg CHBr_3_/kg DMI) and that Asp-Oil would improve cattle performance without effect on cattle health, residues, or meat quality.

## Materials and Methods

The Animal Ethics Committee of the University of New England approved all procedures involved in this experiment (Authority no.: ARA-21-106).

### Animals, diets, and experimental design

A dose–response experimental design was used to evaluate the effect of graded levels of Asp-oil on CH_4_ emissions, rumen fermentation, animal health, and residues over a 59-d finisher-diet feeding period. The design used an incomplete block structure, with two blocks, four treatments, and five replicate animals per treatment. Twenty Angus-cross heifers with a single sire and property of origin (initial age ~15 mo) were transported to the research facility at the University of New England Centre for Animal Research and Teaching, Armidale, NSW, Australia. The heifers were inducted with vaccination (Ultravac® 7‐in‐1, Zoetis, Melbourne Australia and Bovilis MH + IBR, Coopers Animal Health, Macquarie Park, NSW, Australia) and an injectable anthelmintic (Nitromec® Injection, Virbac, Milperra NSW, Australia).

The treatments were three blends of canola oil containing three levels of Asp-Oil—a stock solution of Asp-Oil in a pressed-canola oil base (Sea Forest Ltd, Triabunna, TAS, Australia)—and a blank solvent-extracted canola oil diluent, plus a control of the blank diluent canola oil only. Respective treatment bulk Asp-Oil’s were blended to achieve formulated *Asparagopsis*-derived CHBr_3_ contents of 2,370 mg/kg oil (DM, high Asp-Oil), 1,580 mg/kg oil DM (medium Asp-Oil), 790 mg/kg oil DM (low Asp-Oil), and 0 mg/kg oil DM (control); all supplied at 2.17% of dietary DM. These oil blends were prepared in three batches and stored at 4 °C until mixed into a basal finishing diet (three times per week). The heifers were blocked on initial live weight (block 1 = 335 ± 8.92 kg, *n* = 10; block 2 = 349 ± 8.02 kg, *n* = 10), and within each block randomly allocated to one of the four treatments, in an unbalanced manner (control, *n* = 3 [block 1] and *n* = 2 [block 2]; low Asp-Oil, *n* = 2 [block 1] and *n* = 3 [block 2]; medium Asp-Oil, *n* = 2 [block 1] and *n* = 3 [block 2]; high Asp-Oil, *n* = 3 [block 1] and *n* = 2 [block 2]).

The diet and feeding regime were based on commercial best-practice in the Australian grain-fed cattle industry. The heifers were transitioned from a diet of 100% roughage to 80% tempered barley and 5.0% total lipids as ether extract (DM-basis, Table 1) in a three-ration (Starter, Intermediate I, Intermediate II), 21-d preexperimental adaptation program, during which CH_4_ emissions were measured once for each transition diet (data shown in Supplementary Material). Diets were formulated using Concept 5 software (CFC Tech Services Inc, Staples, MN, USA). The inclusion rate of the treatment oil blends was increased in four equal increments over the transition diets to achieve a target finisher *Asparagopsis*-derived CHBr_3_ content of 0, 17, 34, and 51 mg/kg diet DM in control, low Asp-Oil, medium Asp-Oil, and high Asp-Oil treatments, respectively, (Supplementary Material 1). The diets were initially mixed as a single batch without any oil in a wagon mixer (274-12 Feed Mixer, Rotomix, Dodge City, KS, USA), and then the treatment oils for each treatment group were added separately to sub-batches of the diet, and re-mixed in a ribbon mixer. Fresh diets were mixed three-times per week, and the diets were stored at room temperature until feeding. Mixing time for the oil addition (7 min per sub-batch) was set so that the coefficient of variation of total lipids content of 10 sub-samples of the mix was <5%.

**Table 1.** Proximate composition of feedlot finisher diet fed to Angus heifers during the 59-d feeding period

Item	
Ingredient, % as fed^1^	
Tempered barley	81.0
Oaten chaff	5.5
Whole cottonseed	5.5
Canola oil blend	1.7
Liquid supplement	6.3
Analyzed nutrient composition (DM-basis)^2^	
Dry matter, %	80.1
Organic matter, % DM	94.8
Ash, % DM	5.3
Crude protein, % DM	11.4
Fat, % DM	5.2
Total digestible nutrients, % DM	81.1
Neutral detergent fiber, % DM	28.0
Acid detergent fiber	12.8
Starch, % DM	46.0
Metabolizable energy, Mcal/kg DM	3.15
Net energy_maintenance_, Mcal/kg DM	1.99
Net energy_gain_, Mcal/kg DM	1.34
Calcium, % DM	1.05
Phosphorus, % DM	0.31
Monensin, ppm (formulated)	25.0

^1^Diets were formulated using Concept 5 software.

^2^Chemical testing was performed at NSW DPI Laboratory Services—Wagga Wagga Chemistry Services Laboratory, Wagga Wagga. Total digestible nutrients, metabolizable energy, net energy for maintenance, and net energy for gain were estimated using equations from Heim and Krebs (2018), NASEM (2016), and Galyean et al. (2016).

Throughout the experimental period, the heifers were housed indoors in individual pens, and fed once daily (at 0900 hours for block 1 and 0930 hours for block 2). The block 2 heifers commenced the experiment 1 d after block 1, to enable sampling to occur on the same experimental day in both blocks. Throughout the experimental period, the heifers were fed ad libitum, with each day’s feed offering adjusted to 110% of the previous day’s fresh feed intake. Orts were removed after 23 h and weighed. Individual feed mixes were sampled for DM after each mixing, and feed refusals were sampled for DM daily for individual heifers. Grab samples were collected from each mixer load of the main diet and bulked weekly for nutrient analysis by wet chemistry. All heifers had ad libitum access to clean and fresh water.

### Measurements of emissions

Production of CH_4_ was measured by confining each heifer in an individual open-circuit respiration chamber (*n* = 10, Hegarty et al., 2014) for 23 h on days 13, 27, 41, and 55. The heifers were fed immediately before the chambers were sealed, and at the end of the chamber session, orts were measured and sampled for DM. The concentration of CH_4_ in each chamber’s exhaust air was measured every 9 min with a Servomex Multigas Analyzer (Servomex 4,100 Gas Purity Analyzer, Spectris PLC, Egham, U.K.) calibrated for CH_4_, carbon dioxide (**CO**_**2**_) and oxygen (**O**_**2,**_), and CH_4_ data were corrected for recovery (mean 94.3% ± 3.24%) of a known quantity of pure CH_4_ tested every 4 wk (Hegarty et al., 2014). The point-measures of CH_4_ concentration were averaged for each hour for each chamber, and fitted to a curve of CH_4_ flux for the measurement period, so that area under this curve represented the total CH_4_ production for the measurement period. Methane yield (g CH_4_/kg DMI) was calculated by dividing the CH_4_ production by adjusted DMI, where DMI=0.6×DMI observed in the respiration chambers+0.4×DMI recorded the previous day to account for residual feed in the rumen from the previous day’s feeding.

Ammonia (**NH**_**3**_) emissions in the chambers were recorded using Honeywell ToxiRAE Pro PGM—1,860 in each of the respiration chambers. Before their use, the ToxiRAEs were turned on and made ready for use by calibrating the NH_3_ value to zero in an open environment. The ToxiRAEs were then hung from the ceiling of the respiration chambers to record NH_3_ data (in ppm), every 60 s.

Data from the ToxiRAEs were extracted using ProRAE studio II software (Rae Systems by Honeywell) as.csv files. Daily NH_3_ emissions (g/d) were estimated by calculating the average NH_3_ (ppm) and airflow (calculated from the respiration chamber Servomex) as follows:


$$\begin{array}{l} NH_{3}\;(gd^{-1})=\frac{NH_{3p}\times R\times m}{22.4\times T}\times 17.03 \end{array}$$

where NH_3*p*_ is the average NH_3_ (ppm) while the animals were the chambers, R is the mean air flow rate determined from chamber Servomex corrected, T is the time in chamber (in minutes), *m* is the number of minutes in a day, 22.4 is the volume of 1 mole of any gas at standard temperature and pressure, and 17.03 is the molecular weight of NH_3_.

### Other live animal sampling and analyses

Rumen temperature was monitored continuously using smaXtec rumen boluses (Bolus TX-1442A, smaXtec animal care GmbH, Austria). Immediately after CH_4_ production measurement, the cattle were returned to their individual pens, fed, and 3 h after feeding were weighed for a post-feeding measure of body weight (**BW**) using a single calibrated animal weighing box (Ruddweigh 600 mm Weigh Beam 2,000 kg weighing capacity, Ruddweigh, Guyra, NSW, Australia, on days 14, 28, 42, and 56). Concurrently, samples of rumen fluid, feces, and blood were collected. Rumen fluid was sampled by orogastric intubation, tested for pH (EcoScan Portable pH/ORP meter with TPS pH Sensor) and reduction potential (Mettler Toldeo SevenEasy S20 pH meter with TPS Intermediate Junction Redox Sensor) immediately after sampling, and then subsampled for measurement of volatile fatty acid (**VFA**) profiles and rumen NH_3_ (samples acidified to ~pH 2 with 0.1 M H_2_SO_4_), rumen protozoa enumeration (samples fixed with 4% formal saline [1:4] and stained with Brilliant Green), and dissolved hydrogen and CH_4_ (35 mL rumen fluid transferred into a 50 mL syringe immediately after collection, sealed with three-way Luer Lock stopcock and cooled to room temperature). A venous whole blood sample (10 mL) was analyzed for total leucocytes, neutrophils, and lymphocytes (Abbott-Cell-Dyn Counter 3,700, Abbott Diagnostic Division, Vienna, Austria). A serum sample of venous blood (10 mL) was analyzed for haptoglobin, triiodothyronine (**T3**), thyroxine (**T4**), and vitamin B_12_. A single sample of serum for each animal, pooled from all samples, was analyzed for iodide (**I**^**−**^) and bromide (**Br**^**−**^). A fecal sample was collected from the rectum, frozen within 40 min of collection, and stored at −20 °C. Feces were analyzed for fecal cortisol metabolites (using the MP Biomedical I125 RIA Cortisol Kit [# 07-221106; MP Biomedicals Australia, Seven Hills, NSW, Australia] and the methods of the study by Mayes et al. [2022]), fecal starch, CHBr_3_ (by Analytical Services Tasmania, New Town, TAS, Australia, using a GC-MS purge and trap methodology), I^−^ and Br^−^ content (by Environmental Analysis Laboratory, Lismore, NSW, Australia, using ICP-MS).

### Analysis of dissolved hydrogen and methane in rumen fluid

Extraction of dissolved gas from rumen fluid was performed after the procedure used by Wang et al. (2014). In brief, 5 mL of nitrogen (**N**_**2**_) gas was injected into the rumen fluid sample in the 50 mL syringe connected through the Luer Lock stopcock. The dissolved gases in the rumen fluid were extracted by vigorously shaking the mixture for five minutes and subsequently, the gas was transferred to a 20 mL syringe, and its volume was recorded. The composition of the extracted gas was analyzed by gas chromatography (CP-4900 Micro Gas Chromatograph with Varian Star Workstation software [Varian B. V. 4330 Middleburg, the Netherlands]). Once the gas chromatograph was activated and the pump set on, gas from the 20 mL syringe was steadily injected for 25 s through the gas chromatogram inlet line. A 10 mL reference gas mixture containing 2.17 ± 0.07, 4.48 ± 0.13, 21.36 ± 0.60, and 71.67 ± 0.80 Mol% of hydrogen (**H**_**2**_), CH_4_, N_2_, and CO_2_, respectively, (Air Liquid Australia Limited, North Sunshine, Victoria 3020; product reference # 67258), was used for calibration of the gas chromatography.

The concentration of dissolved H_2_ (***CdH***_***2***_) was calculated based on the formula of Wang et al. (2014) as follows:


$$\begin{array}{l} CdH2=\frac{CgH2}{22.4}\left(\alpha +\frac{Vg}{Vl}\right) \end{array}$$ Eq 1

where *CdH*_*2*_ is the dissolved hydrogen concentration (μM), *CgH*_*2*_ is the gas *H*_*2*_ concentration (*μll*^*−1*^) from the gas phase (obtained from gas chromatogram), 22.4 is the molar volume of H_2_ at 1 atm pressure (22 l mol^−1^), *α* is Bunsen absorption coefficient of H_2_ in distilled water (l.l^−1^) at 39.5 °C calculated to be 0.0166l.l^−1^, *Vg* is the volume of the gas in the syringe after gas extraction, and *Vl* is the volume of the rumen fluid (*l*).

### Chemical analysis of feeds

DM content of feeds offered and refusals was determined on a ~150 g sample by oven drying at 65 °C until there was no change in weight. For mixed diets, a further ~150 g subsample was bulked weekly and analyzed (Table 1) for content of crude protein (AOAC Method 2001.11), acid detergent fiber (AAFCO Method 008.08), neutral detergent fiber (NFTA Method 2.2.2.5), organic matter (ISO 5984:2002[E]), ether extract (AFIA Method 1.14R), starch (AOAC Method 996.11), water-soluble carbohydrates (AFIA Method 1.11A), and digestible dry and organic matter (AFIA Method 1.7R). Total digestible nutrients (TDN) were determined using the following calculation: TDN (%DM) = 93.59—(ADF × 0.936; Heim and Krebs, 2018). TDN was then used to calculate digestible energy (NASEM, 2016) which was subsequently used to calculate metabolizable energy, net energy for maintenance, and net energy for gain (Galyean et al. 2016).

### Slaughter and carcass measurements

All heifers were transported to slaughter on the same day (day 59 [block 1), day 58 [block 2]). The abattoir was 450 km from the experimental facility. The heifers were lairaged overnight and slaughtered in treatment groups. Immediately post-slaughter and dressing, hot standard carcass weight (kg) was determined according to AUS-MEAT carcass standards (AUS-MEAT Limited, 2005). Post-chilling, carcasses were evaluated by two independent accredited Meat Standards Australia (**MSA**) graders (Meat Standards Australia, 2007) for hump height (mm, Meat Standards Australia, 2007) fat color and meat color using the AUS-MEAT color reference standards (AUS-MEAT Limited 2005); MSA marbling score (in chilled carcasses, Meat Standards Australia, 2007); rib fat depth (mm, AUS-MEAT Limited 2005); ossification score (Romans and Ziegler, 1985); ultimate pH (**pHu**); and eye muscle (longissimus thoracis et lumborum) area (eye muscle area, cm^2^, AUS-MEAT Limited 2005). Ultimate pH and loin temperature were measured in the rib eye muscle (longissimus thoracis et lumborum) at the time of carcass grading. Carcass grading data was then used to calculate an MSA index value as described by McGilchrist et al. (2019) to estimate the predicted eating quality of each carcass.

The rumens of all the cattle were collected immediately post-slaughter, cleaned, and examined for gross pathology by trained veterinary pathologists as described by Jonsson et al. (2020). In brief, subjective scores were ascribed for papillae color and shape, and indication of pathological alterations, to the ventral sac of the rumen. A sample of normal and lesioned (where evidenced) rumen walls was taken for histological analysis (Jonsson et al., 2020). Tissue samples included representative samples of the rumen wall and any significant findings from the macroscopic (i.e., gross) examination.

### Consumer sensory analysis of eating quality

At boning, portions of the striploin (*M. longissimus dorsi lumborum*, at muscle positions A1, A2, and P4) were cut and vacuum packed for sensory testing and stored at 1 to 3 °C until fabrication of 25 mm test steaks according to the protocols of Watson et al. (2008). Five sample steaks were prepared from each sample striploin, vacuum sealed, chilled, and stored at −20 °C until sensory testing. Shear force samples were also fabricated from the posterior end of the sensory striploin (P3) to ensure that results were taken in between sensory samples.

In accordance with MSA protocols (Meat Standards Australia, 2008; Watson et al., 2008), samples were evaluated by a panel of 60 untrained consumers, with 10 consumers evaluating each of the 42 samples and all consumers being served seven samples. The MSA consumer taste panel used untrained consumers to score meat samples for tenderness, juiciness, like flavor, and overall acceptability. While the consumers were untrained, they were screened to include only people who preferred steak cooked to medium doneness, ate beef at least once a fortnight and were aged between 18 and 70 yr old.

Frozen steaks were thawed and then cooked to achieve a medium degree of doneness using a double-sided clam shell SilexTM S-Tronic 161K grill. After cooking, steaks were rested, halved, and served to the consumer panelists who individually scored samples by making a mark along a 100 mm scale for ‘tenderness’, anchored by the words ‘not tender’/’very tender’; ‘juiciness’, anchored by the words ‘not juicy’/’very juicy’; and ‘like flavor’ and ‘overall acceptability’, both anchored by the word ‘dislike extremely’/’like extremely’ for both. The consumer panel also rated the samples for eating quality based on the following scoring system: unsatisfactory (two stars), good every day (three stars), better than every day (four stars) the premium eating quality (five stars). The four sensory scores were weighted to provide a single meat quality score (**MQ4**) based on a linear discriminant function to provide the best allocation of samples to the four quality grades (Polkinghorne, 1999).

### Data processing and statistical analysis

All data from two heifers were removed from the analyzed dataset due to chronic health problems: one heifer in the control group (Tag #19) had a behavioral pattern of rapid meal consumption, which likely caused a chronic, sub-acute acidosis (mean pH 5.94) which showed signs of becoming acute (i.e., diarrhea) from day 31, and on day 46 the decision was made to remove her from the experiment. One heifer in the High Asp-Oil treatment group (Tag #76) maintained low intakes (frequently under 2 kg/d) from day 7 of the adaptation period onwards and subsequently experienced weight loss, indicating maladaptation to the grain diet; however, she showed no other behavioral signs or symptoms of acidosis or illness during the experimental period. This heifer’s rumen pH was a mean of 5.54, which is a level commonly used to diagnose subacute and acute acidosis (Nagaraja and Lechtenberg, 2007). Rumen NH_3_ levels were also indicative of rumen acidosis (Herrero et al., 2014), with levels significantly greater than the rest of the cohort (156 to 721 mg/L vs 0 to 131 mg/L). At slaughter, the gross morphology of her rumen wall indicated a chronic, sub-acute acidosis. Full data from these animals is provided and identified by Tag ID in Supplementary Material.

DMI was calculated using daily records for fresh weight of feed offered and refusals, weekly dry matter content (sampled at mixing) for each diet for feed offered, and daily samples of refusals for each heifer, bulked weekly. Individual animal measurements of intake were removed from the DMI dataset on days where fecal contamination of the feed trough overnight caused refusals to exceed 1 kg fresh weight.

Liveweight gain was calculated by the slope of the linear regression of pre-feeding liveweight measurement (days 0 to 59). Gain:feed was calculated as follows: $GF_{ij}=LW_{ij}-\;LW_{i(\;j-1)}/\sum DM{I_{(\;j-1\ldots j}}_{)}$ where GF_ij_ is Gain:Feed for the i^th^ animal in the j^th^ period, LW_ij_ is liveweight for the i^th^ animal in the j^th^ period, and DMI_(j-1…j)_ is dry matter intake for the j^th^ period.

Repeated measures of CH_4_, rumen function, blood hematology, and performance were analyzed with a mixed model linear regression, with block and animal as random effects, and including the interaction of Asp-Oil dose with sampling day as follows: $Y_{ijkl}=\mu +\;A_{j}+\;P_{j}+AP_{ij}+b_{k}+e_{ijkl}$ where μ is the overall mean, A_i_ is the effect of the i^th^ ASP dose (control, low, medium, and high)—orthogonal contrasts, P_j_ is the effect of the j^th^ sampling day (j = 1, …, 59), AP_ij_ is the interaction between the i^th^ Asp-Oil dose and the j^th^ day, b_k_ is the effect of the k^th^ block, and e_ijkl_ is the random error associated with the l^th^ repetition of the i^th^ ASP dose in the j^th^ sampling period in block k ~ N(0,σ^2^_e_).

Several models of variance–covariance were tested for each response, and the model of best fit was chosen by the lowest Akaike Information Criterion value. For methane emissions data, the residuals were not normally distributed, so a log + 0.05 transformation was applied to the data before the linear regression. As the back-transformation produced distorted means, least squared means of methane emissions were reported from the untransformed data, but orthogonal contrasts from the transformed data.

Post-slaughter measures of carcass performance and residues, and pooled serum residue samples were analyzed with a mixed model linear regression with block as a random effect: $Y_{ijkl}=\mu +\;A_{j}+b_{k}+\;e_{ijkl}$

For consumer sensory scores, each dependent variable (MQ4, tenderness, juiciness, flavor, and overall liking) were analyzed in a linear mixed effects model with treatment and sample position from the striploin (anterior, center, and posterior) as fixed effects and carcass number as a random intercept.

Longitudinal changes in circadian patterns of rumen temperature were analyzed with the package *cosinoRmixedeffects* (Hou et al., 2021) to estimate the non-linear parameters of midline statistic of rhythm (**MESOR**, a function that depends on the rhythm-adjusted mean), amplitude (half the extent of variation within a day) and acrophase (the time of overall high values recurring in each day, relative to the overall mean). Counts of protozoa were left-skewed and zero-inflated, and so these were analyzed with a Kruskal–Wallis test, with Dunn’s test for multiple comparisons of groups. All other parametric data were analyzed using the *lme4* (Bates et al., 2015) package of R Statistical Software. Least-squares means, linear, and quadratic orthogonal contrasts were computed with the *emmeans* package (Lenth, 2020), using the Holm-Bonferroni adjustment for multiple treatment groups.

## Results

### Methane emissions and rumen function

**Figure 1.**
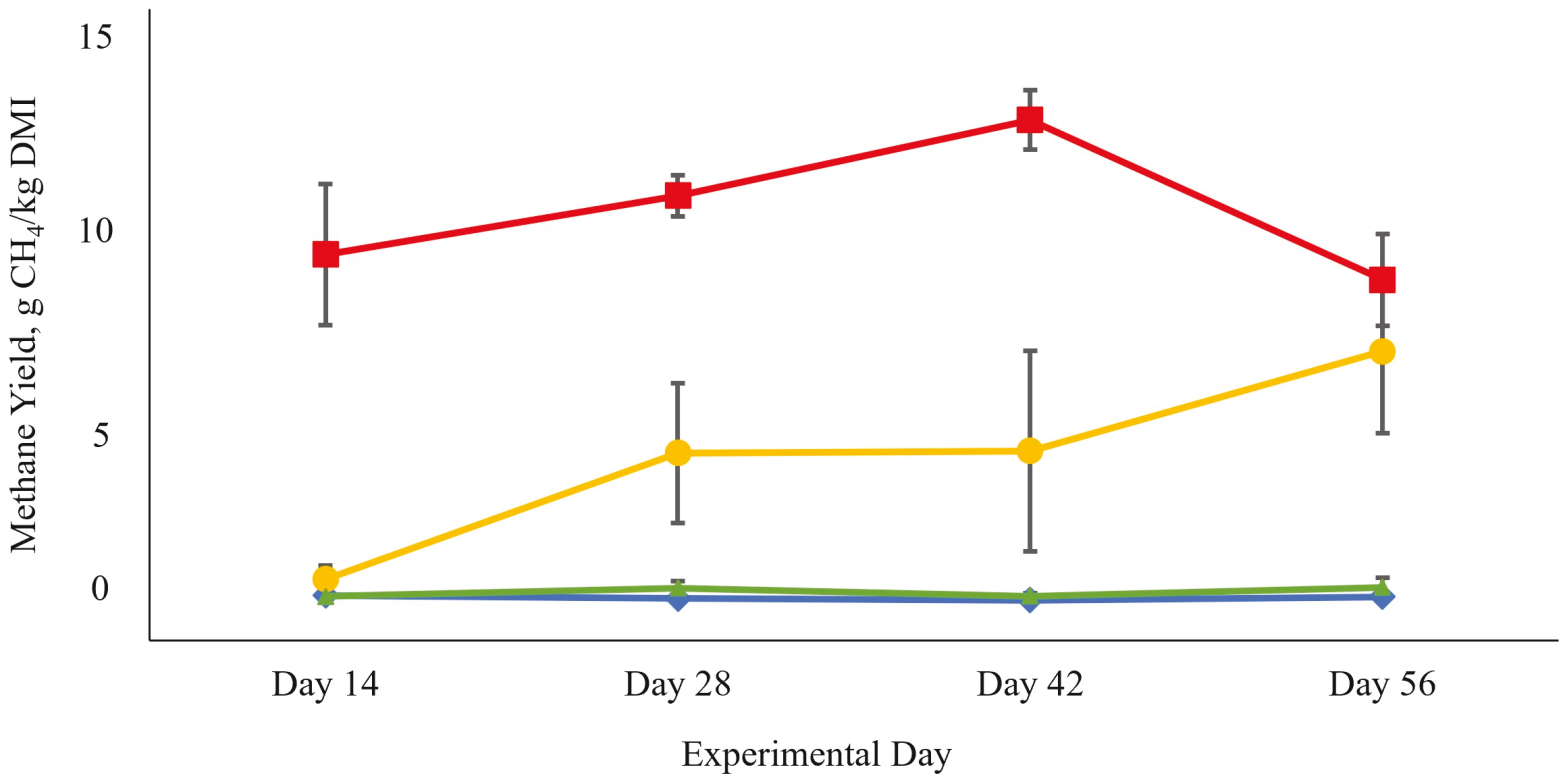
Methane yield (g CH_4_/ kg dry matter [DM]) of Angus heifers (observed mean ± s.e.; *N* = 18) fed increasing levels of *Asparagopsis* bioactives stabilized in a canola oil carrier (Asp-Oil) during the four measurement times during the finishing period ( Squares, control, 0 mg CHBr_3_/kg DM; Circles, low Asp-Oil, 17 mg CHBr_3_/kg DM; Triangles, medium Asp-Oil, 35 mg CHBr_3_/kg DM; Diamonds, high Asp-Oil, 51 mg CHBr_3_/kg DM).

The addition of Asp-Oil resulted in a significant linear reduction in CH_4_ production (g/d, *P* < 0.001) and yield (g/kg DMI, *P* < 0.001). Compared to the control, the CH_4_ yield reductions for each treatment group were 64%, 98%, and 99% for the low, medium, and high Asp-Oil inclusions, respectively (Table 2). For the medium and High Asp-Oil groups, CH_4_ production and yield did not differ from zero. The low Asp-Oil group demonstrated an increase in CH_4_ production and yield after day 12 of the experiment, such that by day 56 it did not differ from the control treatment (*P* = 0.371 for CH_4_ production, and *P* = 0.330 for CH_4_ yield), whereas the suppression of CH_4_ production and yield persisted throughout the experimental period for both the medium and High Asp-Oil groups (Figure 1).

**Table 2.** Least-squares means (± standard error) of enteric methane (CH_4_) emissions, post-feeding rumen fermentation parameters, and intake (dry matter intake) in respiration chambers of Angus heifers fed canola oil of increasing bromoform (CHBr_3_) content, during four open-circuit respiration chambers runs

	Asp-Oil treatment^1^				*P*-value^2^			
	Control	Low	Medium	High	T		D	T × D
					Linear	Quadratic		
DMI^3^, kg	10.1 ± 0.70	9.29 ± 0.66	8.85 ± 0.66	9.60 ± 0.728	0.441	0.188	0.160	0.310
CH_4_, g/d	129 ± 11.9	43.8 ± 10.9	10.0 ± 10.9	1.4 ± 13.4	<0.001	0.172	0.080	0.040
CH_4_ yield, g/kg DMI	9.80 ± 0.94	3.50 ± 0.86	0.20 ± 0.86	0.10 ± 1.1	<0.001	0.194	0.080	0.015
Ammonia emissions, g/d	5.20 ± 1.47	7.10 ± 1.35	6.40 ± 1.35	8.50 ± 1.65	0.849	0.553	0.730	0.620
Rumen pH	6.47 ± 0.18	6.97 ± 0.16	6.52 ± 0.16	6.35 ± 0.19	0.341	0.262	0.892	0.003
Redox potential	−98.6 ± 39.1	−98.1 ± 37.7	−102 ± 37.7	−100 ± 39.1	1.000	1.000	<0.001	0.887
Total dissolved gases, mL/g rumen fluid	11.3 ± 1.27	9.10 ± 1.23	10.9 ± 1.31	10.4 ± 1.17	1.000	1.000	0.340	0.160
Dissolved hydrogen, µM	90.0 ± 394	689 ± 370	1392 ± 413	1506 ± 336	0.027	0.778	0.720	0.150
Dissolved CH_4,_ mM	3.18 ± 0.42	1.05 ± 0.45	0.12 ± 0.48	0.05 ± 0.43	0.017	0.850	0.140	0.570
Rumen ammonium, mg N/L	42.9 ± 11.5	47.4 ± 10.5	42.8 ± 10.5	30.3 ± 12.9	1.000	1.000	< 0.001	0.372
Total VFA, mmol/L	67.6 ± 7.32	60.3 ± 6.75	70.8 ± 6.75	66.9 ± 7.89	1.000	1.000	0.544	0.087
Acetate, mol/100 mol	55.8 ± 1.84	52.5 ± 1.69	52.3 ± 1.69	51.2 ± 2.07	0.378	1.000	0.476	0.913
Propionate, mol/100 mol	20.2 ± 2.65	22.8 ± 2.43	22.6 ± 2.43	22.1 ± 2.97	1.000	1.000	0.678	0.688
Butyrate, mol/100 mol	19.3 ± 2.71	19.8 ± 2.49	19.4 ± 2.49	22.3 ± 3.04	1.000	1.000	0.933	0.191
Acetate:propionate	2.9 ± 0.33	2.6 ± 0.30	2.7 ± 0.30	2.4 ± 0.37	1.000	1.000	0.611	0.688

^1^Asp-oil levels were control = 0 mg CHBr_3_/kg oil DM, *n* = 4; low = 791 mg CHBr_3_/kg oil DM, *n* = 5; Medium = 1,591 mg CHBr_3_/kg oil DM, *n* = 5; high = 2,389 mg CHBr_3_/kg oil DM, *n* = 4, included in the diet at 2.17% DM.

^2^T: Asp-Oil treatment; D: day; T × D: treatment × day. The main effect of T was decomposed into linear and quadratic orthogonal contrasts, using the Holm-Bonferroni adjustment for multiple comparisons.

^3^DMI accounts for the mean intake in respiration chamber (60%) and intake of the previous day (40%).

Methane dissolved in rumen fluid followed a similar pattern to eructated CH_4_, with all Asp-Oil treatments significantly (*P* = 0.017) reducing dissolved CH_4_. Hydrogen dissolved in rumen fluid increased linearly (*P* = 0.027) with increasing Asp-Oil inclusion. Despite the accumulation of H_2_ in rumen fluid, pH and reduction potential were not affected by Asp-Oil treatment (*P* = 0.341, *P* = 1.00, and *P* = 1.00, respectively), although reduction potential declined significantly in all groups on the last measurement day. There was no effect of Asp-Oil treatment on total VFA or molar proportions of VFA, including acetate:propionate ratio. There was no effect of Asp-Oil on enumerated protozoa (Table 3).

**Table 3.** Protozoa counts (median [min, max]) of Angus heifers fed increasing levels of *Asparagopsis* bioactives stabilized in a canola oil carrier (Asp-Oil) during the four measurement times during the overall feeding period

Item	Asp-Oil treatment^1^					
	Control	Low	Medium	High	Χ^2^	*P*
Total protozoa, × 10^3^/mL	0.156 [0, 23.1]	0.023 [0, 57.8]	0.008 [0, 1.38]	0 [0, 18.6]	2.824	0.420
Large holotrich, × 10^3^/mL	0 [0, 0]	0 [0, 0]	0 [0, 0.19]	0 [0, 0]	5.273	0.153
Small holotrich, × 10^3^/mL	0 [0, 0.03]	0 [0, 0.09]	0 [0, 0.91]	0 [0, 0.06]	1.971	0.579
Entodiniomorphs, × 10^3^/mL	0.500^a^ [0, 23.1]	0.078^ab^ [0, 57.8]	0.016^b^ [0, 7.1]	0^b^ [0, 18.6]	3.781	0.286

^1^Asp-oil levels were control = 0 mg CHBr_3_/kg oil DM, *n* = 4; low = 791 mg CHBr_3_/kg oil DM, *n* = 5; medium = 1,591 mg CHBr_3_/kg oil DM, *n* = 5; high = 2,389 mg CHBr_3_/kg oil DM, *n* = 4, included in the diet at 2.17% DM; Rumen fluid samples collected on four occasions (day 14 to 56).

### Animal performance and health

All heifers started the transition period (*P* = 0.912) at similar BW (Table 4). Supplementation with Asp-Oil during the 59-d finisher period did not affect BW at the end of the adaptation period, final BW, DMI, average daily gain (**ADG**) nor gain to feed ratio (**G:F**; *P* ≥ 0.739; Table 4).

**Table 4.** Least-squares means of intake (dry matter intake), live weight (LW) gain, and gain:feed ratio in Angus heifers fed canola oil of increasing CHBr_3_ content

	Asp-Oil treatment^1^				*P*-value^2^	
	Control	Low	Medium	High	Linear	Quadratic
DMI^3^, kg/d	9.72 ± 0.86	8.87 ± 0.83	8.73 ± 0.83	9.90 ± 0.87	1.000	0.211
DMI^3^, g/kg BW	23.7 ± 1.53	22.4 ± 1.46	21.8 ± 1.46	24.3 ± 1.57	1.000	0.271
Initial BW^4^, kg	346 ± 5.96	345 ± 5.74	343 ± 5.74	336 ± 6.10	0.912	0.535
Adaptation period end BW^5^, kg	373 ± 9.84	364 ± 9.69	369 ± 9.69	370 ± 9.91	0.960	0.960
Final LW^5^ (day 58/59), kg	457 ± 15.5	439 ± 15.0	440 ± 15.0	456 ± 15.7	0.973	0.208
BW gain^4^^,^^6^, kg/day	1.52 ± 0.14	1.31 ± 0.19	1.31 ± 0.19	1.45 ± 0.19	0.739	0.739
Average daily gain^4^^,^^7^, kg/day	1.36 ± 0.17	1.24 ± 0.16	1.26 ± 0.16	1.39 ± 0.19	1.000	1.000
Gain:Feed^4^^,^^8^, kg/kg	0.16 ± 0.02	0.15 ± 0.02	0.14 ± 0.02	0.16 ± 0.03	1.000	1.000

^1^Asp-oil levels were control = 0 mg CHBr_3_/kg oil DM, *n* = 4; low = 791 mg CHBr_3_/kg oil DM, *n* = 5; medium = 1,591 mg CHBr_3_/kg oil DM, *n* = 5; high = 2,389 mg CHBr_3_/kg oil DM, *n* = 4, included in the diet at 2.17% DM.

^2^The main effect of Asp-Oil treatment was decomposed into linear and quadratic orthogonal contrasts, using the Holm-Bonferroni adjustment for multiple comparisons.

^3^DMI measured daily.

^4^Liveweight measured pre-feeding.

^5^Liveweight measured post-feeding.

^6^Liveweight gain (kg/d) determined by regression of live weight on time (days 0 to 56).

^7^Arithmetic mean of average daily gain for four measurement periods (days 0 to 56).

^8^Mean of period live weight change/feed intake for four measurement periods (days 0 to 56).

The inclusion of Asp-Oil did not affect serum thyroid hormone, vitamin B_12_, hemoglobin, or haptoglobin concentrations, fecal glucocorticoid metabolite concentration, or parameters of rumen temperature rhythm (Table 5). Differentiation of plasma cells was not affected by Asp-Oil, except for linear increases in neutrophil (*P *= 0.007) and platelet (*P* = 0.016) counts with increasing Asp-Oil (Table 5). Scores of rumen wall condition, papillae color, and shape were not affected by Asp-Oil. In histological samples of the rumen wall, focal or multifocal small aggregates of lymphocytes, plasma cells, and neutrophils in the submucosa, as well as areas with fewer or shorter papillae were present in all groups, including the control group. In the control group, three rumens were considered grossly and histologically normal; one demonstrated focal areas with parakeratosis and intraepithelial pustules; and displayed chronic ruminitis with fibrosis. One heifer in the control group was removed from the study due to persistent ruminal acidosis. In the low Asp-Oil treatment group, two rumens were considered grossly and histologically normal; and three out of five rumens contained mild microscopical parakeratosis without evidence of gross lesions. In the medium Asp-Oil group, one rumen was grossly and histologically normal; two demonstrated mild parakeratosis; and one was considered with severe parakeratosis; there was ulceration in one out of the five rumens; and mild scarring in two out of five rumens. In the high Asp-Oil group, two out of four rumens were considered grossly and histologically normal; and two showed mild histological microscopical parakeratosis.

**Table 5.** Measures of health and welfare (mean ± standard error) in Angus heifers fed increasing levels of *Asparagopsis* bioactives stabilized in a canola oil carrier (Asp-Oil) for a 59-d finisher feeding period

	Asp-Oil treatment^1^				*P*-value^2^	
	Control	Low	Medium	High	Linear	Quadratic
Serum thyroxine, ng/mL	2.40 ± 0.44	2.14 ± 0.42	2.56 ± 0.42	3.14 ± 0.46	0.351	0.504
Serum triiothyronine, ng/mL	69.5 ± 9.80	71.2 ± 9.38	76.8 ± 9.32	85.6 ± 10.1	0.356	1.000
Fecal glucocorticoid metabolites, ng/g	0.81 ± 0.18	0.95 ± 0.22	0.99 ± 0.22	1.16 ± 0.19	1.000	1.000
Serum vitamin B_12_, *p*g/mL	65.5 ± 14.9	61.5 ± 14.3	47.1 ± 13.7	76.9 ± 16.7	0.928	0.928
Rumen wall						
Papillae color score^3^	1.0 ± 0.21	0.8 ± 0.19	0.8 ± 0.19	0.8 ± 0.23	0.730	—
Papillae shape score^4^	0.5 ± 0.48	0.7 ± 0.44	0.4 ± 0.44	1.3 ± 0.52	0.490	—
Ventral sac damage score^3^	0.6 ± 0.80	0.5 ± 0.76	1.5 ± 0.76	0.5 ± 0.81	0.410	—
Rumen temperature, mean (95% CI)						
MESOR^5^, °C	39.2 (39.06 to 39.40)	39.1 (39.00 to 39.31)	39.2 (39.04 to 39.37)	39.1 (38.95 to 39.28)	—	—
Amplitude^6^, °C	0.26 (0.257 to 0.269)	0.26 (0.256 to 0.267)	0.22 (0.216 to 0.228)	0.29 (0.286 to 0.297)	—	—
Acrophase^7^, h	−5.98 (−6.00 to −5.96)	−0.01 (−3.57 to 5.71)	−6.17 (−6.20 to −6.15)	−6.13 (−6.15 to −6.11)	—	—
Haptoglobin, mg/L	292 ± 72.8	475 ± 69.8	313 ± 67.9	338 ± 76.6	0.938	0.481
Hematocrit, %	32.5 ± 1.38	32.0 ± 1.26	32.0 ± 1.26	31.8 ± 1.55	1.000	1.000
Hemoglobin, g/dL	11.5 ± 0.48	11.4 ± 0.44	11.3 ± 0.44	11.1 ± 0.53	1.000	1.000
Mean corpuscular hemoglobin, pg	15.9 ± 0.67	16.2 ± 0.62	15.6 ± 0.62	15.4 ± 0.76	1.000	1.000
Mean corpuscular hemoglobin concentration, g/dL	35.6 ± 0.28	25.6 ± 0.25	35.3 ± 0.25	34.8 ± 0.31	0.151	0.699
Mean corpuscular volume, fL	44.7 ± 2.10	45.6 ± 1.93	43.9 ± 1.93	44.3 ± 2.36	1.000	1.000
Cellular differentiation of plasma						
Leucocytes, × 10^6^/mL	10.4 ± 0.79	10.8 ± 0.73	10.5 ± 0.73	11.2 ± 0.89	1.000	1.000
Neutrophils, × 10^6^/mL	3.11 ± 0.33	3.25 ± 0.30	3.81 ± 0.30	4.8 ± 0.37	0.007	0.477
Lymphocytes, × 10^6^/mL	6.04 ± 0.60	6.33 ± 0.55	5.23 ± 0.55	5.36 ± 0.68	0.846	0.901
Monocytes, × 10^6^/mL	0.85 ± 0.10	0.74 ± 0.09	0.80 ± 0.09	0.81 ± 0.10	1.000	1.000
Eosinophils, × 10^6^/mL	0.23 ± 0.11	0.27 ± 0.10	0.45 ± 0.10	0.14 ± 0.13	0.849	0.482
Basophils, × 10^6^/mL	0.14 ± 0.04	0.14 ± 0.04	0.13 ± 0.04	0.11 ± 0.04	0.915	1.000
Erythrocyte, × 10^6^/mL	7.28 ± 0.26	7.06 ± 0.24	7.27 ± 0.24	7.19 ± 0.29	1.000	1.000
Platelets, × 10^6^/mL	617 ± 78.0	554 ± 74.8	705 ± 74.8	811 ± 79.3	0.016	0.203

^1^Asp-Oil levels were control = 0 mg CHBr_3_/kg oil DM, *n* = 4; low = 791 mg CHBr_3_/kg oil DM, *n* = 5; medium = 1,591 mg CHBr_3_/kg oil DM, *n* = 5; high = 2,389 mg CHBr_3_/kg oil DM, *n* = 4, included in the diet at 2.17% DM

^2^The main effect of Asp-Oil treatment was decomposed into linear and quadratic orthogonal contrasts, using the Holm-Bonferroni adjustment for multiple comparisons.

^3^Scored on 1 to 5 scale.

^4^Scored on 1 to 4 scale.

^5^Midline Statistic Of Rhythm, a non-linear function that depends on the rhythm-adjusted mean.

^6^half the extent of variation within a day.

^7^the time of overall high values recurring in each day, relative to the overall mean; blood and feces collected on four occasions (days 14 to 56).

### Mass balance of bromoform, bromide, and iodide

Bromoform could not be detected in any samples of feces, kidney, liver, fat or striploin (limit of detection < 2 mg CHBr_3_/kg; data not shown), and was not analyzed in serum. The low, medium, and high Asp-Oils contributed 55, 111, and 166 mg Br^-^/kg total dietary DM, and 0.78, 1.58, and 2.36 mg I^−^/kg total dietary DM, respectively, from the stock Asp-Oil, apart from that supplied from the basal diet and diluent oil. Serum I^−^ concentration declined from control levels in the high Asp-Oil treatment only (*P* = 0.021, Table 6). Serum Br^−^ content, meanwhile, increased linearly as Asp-Oil content increased (*P* < 0.001, Table 6). Bromide and I^−^ concentrations were below detectable limits in all samples of fat (Table 6). The sampling site with the greatest concentration of Br^−^ and I^−^ was the kidney, in all treatments (Table 6). In kidney, liver, and striploin, there was a linear response of Br^−^ concentration to increasing Asp-Oil level (*P* < 0.001), but no effect on I^−^ concentration (Table 6). The greatest level detected for I^−^ was 0.3 mg/kg as measured in two kidney samples in the low Asp-Oil treatment, two in the medium Asp-Oil treatment, and one in the high Asp-Oil treatment. In feces, I^−^ and Br^−^ concentrations increased numerically with Asp-Oil inclusion; however, were not statistically significant (*P* = 0.181) and (*P* = 1.00), respectively (Table 6). There was a significant negative effect of time on fecal I^−^ and Br^−^ concentration, declining with days on feed in all treatment groups (*P* < 0.001, data not shown).

**Table 6.** Iodide and bromide concentrations (least squares mean ± s.e.) in serum (pooled samples), feces (repeated measures), kidney, liver, fat, and striploin of Angus heifers fed increasing levels of Asparagopsis bioactives stabilized in a canola oil carrier (Asp-Oil)

	Asp-Oil treatment^1^				*P*-value	
	Control	Low	Medium	High	Linear	Quadratic
Iodide						
Serum, mg/L	1.17 ± 1.25	1.42 ± 0.25	1.16 ± 0.25	0.86 ± 0.25	0.021	0.018
Feces, mg/kg	0.45 ± 0.05	0.51 ± 0.04	0.68 ± 0.04	0.80 ± 0.06	181	0.181
Kidney, mg/kg	0.16 ± 0.03	0.24 ± 0.03	0.20 ± 0.03	0.24 ± 0.03	0.797	0.126
Liver, mg/kg	<0.1	<0.1	<0.1	<0.1	−	−
Fat, mg/kg	<0.1	<0.1	<0.1	<0.1	−	−
Striploin, mg/kg	<0.1	<0.1	<0.1	<0.1	−	−
Bromide						
Serum, mg/L	18.7 ± 1.33	26.2 ± 1.22	37.2 ± 1.22	41.6 ± 1.49	<0.001	0.285
Feces, mg/kg	247 ± 22.1	256 ± 19.6	243 ± 19.6	250 ± 22.5	1.000	1.000
Kidney, mg/kg	12.0 ± 0.71	17.2 ± 0.55	21.6 ± 1.50	25.6 ± 1.63	0.039	0.039
Liver, mg/kg	6.0 ± 0.41	8.9 ± 0.20	12.1 ± 0.32	12.6 ± 0.85	<0.001	0.032
Fat, mg/kg	<5.0	<5.0	<5.0	<5.0	−	−
Striploin, mg/kg	<5.0	<5.0	<5.0	6.00 ± 0.48	−	−

^1^Limit of detection of iodide was 0.1 mg/kg and bromide was 5.0 mg/kg.

### Carcass quality and meat sensory characteristics

There was no effect of treatment on carcass weight, grading, or shear force (*P* > 0.10, Table 7). Similarly, the sensory evaluations confirmed that there was no significant difference for any of the eating quality traits in any of the treatment groups compared to the control (Table 8).

**Table 7.** Responses (least squared means ± s.e.) of hot standard carcass weight (HSCW), dressing percentage, P8 fat depth (P8 fat), rib fat depth (Rib fat), eye muscle area (EMA), Meat Standards Australia marbling scores (MSA Marbling), ossification score (Ossification), ultimate pH (pHu), Meat Standards Australia index (MSA index) in Angus heifers (*N* = 18) fed increasing levels of *Asparagopsis* bioactives stabilized in a canola oil carrier (Asp-Oil) after an 59 d feedlot finishing period

	Asp-Oil treatment^1^				*P*-value	
	Control	Low	Medium	High	Linear	Quadratic
HSCW, kg	229 ± 7.43	222 ± 7.18	220 ± 7.18	234 ± 7.54	0.213	0.502
Dressing percentage, %	49.6 ± 0.60	50.7 ± 0.61	50.2 ± 0.57	49.7 ± 0.57	0.936	0.220
P8 fat, mm	10.0 ± 1.15	9.40 ± 1.06	9.80 ± 1.06	9.25 ± 1.30	1.000	1.000
Rib fat, mm	6.50 ± 0.96	5.60 ± 0.88	4.20 ± 0.88	5.50 ± 1.08	0.406	1.000
EMA, cm^2^	63.8 ± 2.23	66.7 ± 2.11	67.5 ± 2.11	71.9 ± 2.30	0.409	0.086
MSA marbling score	308 ± 21.4	288 ± 19.6	288 ± 19.6	310 ± 29.9	0.871	1.000
AUSMEAT marbling score	0.50 ± 0.27	0.40 ± 0.25	0.40 ± 0.25	0.80 ± 0.31	1.000	1.000
AUSMEAT fat color score	0.00 ± 0.20	0.30 ± 0.19	0.20 ± 0.19	0.30 ± 0.23	1.000	1.000
AUSMEAT meat color score	3.00 ± 0.48	3.30 ± 0.45	3.50 ± 0.45	3.10 ± 0.49	1.000	1.000
Ossification score	145 ± 5.95	138 ± 5.45	140 ± 5.45	140 ± 6.67	1.000	1.000
pH_u_	5.53 ± 0.03	5.57 ± 0.03	5.64 ± 0.03	5.62 ± 0.03	0.110	0.835
MSA index	55.6 ± 6.48	50.3 ± 6.00	39.8 ± 6.00	51.8 ± 6.91	0.235	1.000
Shear force, kg	5.53 ± 1.05	4.96 ± 0.97	5.44 ± 0.97	5.09 ± 1.14	1.000	1.000

^1^Asp-oil levels were control = 0 mg CHBr_3_/kg oil DM; low = 791 mg CHBr_3_/kg oil DM; medium = 1,591 mg CHBr_3_/kg oil DM; high = 2,389 mg CHBr_3_/kg oil DM, included in the diet at 2.17% DM.

**Table 8.** Consumer sensory scoring of eating quality of samples from the striploin (*M. longissimus dorsi lumborum)* of Angus heifers (*N* = 18) fed canola oil of increasing bromoform (CHBr_3_) content

	Tenderness		Juiciness		Flavor		Overall liking		MQ4	
Predictors	Estimate	*P*	Estimate	*P*	Estimate	*P*	Estimate	*P*	Estimate	*P*
Asp-oil treatment^1^										
(Intercept)	54.2	<0.001	58.9	<0.001	58.2	<0.001	57.4	<0.001	56.8	<0.001
Control	*Reference*		*Reference*		*Reference*		*Reference*		*Reference*	
Low	−4.47	0.638	−1.77	0.773	0.33	0.958	−1.64	0.835	−1.91	0.802
Medium	0.79	0.934	1.39	0.82	3.87	0.533	1.98	0.800	2.13	0.780
High	−1.65	0.869	−1.96	0.762	0.85	0.896	−1.45	0.861	−0.87	0.914
*Muscle position*										
A1	*Reference*		*Reference*		*Reference*		*Reference*		*Reference*	
A2	−2.36	0.375	−1.01	0.674	−2.89	0.237	−2.41	0.294	−2.4	0.287
P4	−3.09	0.246	−3.37	0.162	−2.38	0.329	−2.88	0.210	−2.84	0.208
*Random effects*										
ơ^2^	62.42		50.76		52.33		46.35		44.64	
Ʈ_00_	178 _carcass_no_		65.7 _carcass_no_		66.9 _carcass_no_		120 _carcass_no_		113 _carcass_no_	
ICC	0.74		0.56		0.56		0.72		0.72	
N	18 _carcass_no_		18 _carcass_no_		18 _carcass_no_		18 _carcass_no_		18 _carcass_no_	
Observations	54		54		54		54		54	
Marginal *R*^*2*^/conditional *R*^*2*^	0.025/0.747		0.034/ 0.579		0.034/0.576		0.023/0.727		0.025/0.723	

^1^Asp-oil levels were control = 0 mg CHBr_3_/kg oil DM, *n* = 4; low = 791 mg CHBr_3_/kg oil DM, *n* = 5; medium = 1,591 mg CHBr_3_/kg oil DM, *n* = 5; high = 2,389 mg CHBr_3_/kg oil DM, *n* = 4, all included in the diet at 2.17% DM.

Estimated coefficients should be interpreted relative to the baseline level, which for treatment was the control sample and for the position was the anterior position (A1).

## Discussion

The present experiment has demonstrated that when compared to previous research using its counterpart FD-Asp, Asp-Oil is equally as efficacious in mitigating enteric CH_4_ emissions from grain-fed beef cattle. Maximal CH_4_ mitigation (98% to 99%) was achieved from the medium and high Asp-Oil treatments, supplying 34 and 51 mg CHBr_3_/kg DM, respectively. Previously in beef finisher diets, dietary inclusion of FD-Asp supplying *Asparagopsis*-derived CHBr_3_ at rates of 24 mg/kg DM (Kinley et al., 2020) and 35 mg/kg DM (Roque et al., 2021) reduced CH_4_ yield by 98% and 70%, respectively, compared to the control. Similar dietary CHBr_3_ inclusions were supplied by the present Medium Asp-Oil treatment of 34 mg/kg DM, which achieved a CH_4_ yield reduction of 98% compared to control (9.8 g CH_4_/kg DMI). From this consistent demonstration in the study by Kinley et al. (2020), Roque et al. (2021), and the current study it is apparent that it is the secondary metabolites produced by *Asparagopsis* (Paul et al., 2006), which are dominated by CHBr_3_ (Machado et al., 2016), that provide the CH_4_ mitigation effect. Furthermore, CH_4_ mitigation can be estimated by accounting for the inclusion rate of CHBr_3_ delivered as Asp-Oil in high-grain feedlot diets. That said, there are notable differences in the mitigation measured in these three studies. The present study and Kinley et al. (2020) both used the gold standard respiration chamber technique to monitor CH_4_ emissions while the Roque et al. (2021) study employed the GreenFeed technique (C-Lock Inc., Rapid City, South Dakota). The former two studies both achieved virtual elimination of CH_4_ yield while the latter demonstrated significant, but comparably lower, antimethanogenic efficacy. Considering the similarity in the feed regime and formulation this may suggest an underestimation of efficacy in this scenario using the GreenFeed system. Further investigation with comparative evaluation of the techniques and limitations of the measurement mechanisms is warranted.

At the 34 and 51 mg CHBr_3_/kg DMI inclusions, the suppression of CH_4_ was persistent over the full period of monitoring. However, concomitant with decreasing antimethanogenic efficacy, an increase in CH_4_ emissions and yield in the low Asp-Oil group (17 mg CHBr_3_/kg DMI) was evident from days 37 to 55 of the experimental period. This suggests that adaptation by the rumen microbiome may be a feature with low-level Asp-Oil CHBr_3_ inclusion that advanced late in the feeding period in this study. Cowley et al. (2023) reported a study of long-fed feedlot Wagyu steers that provides confirmation that emissions reductions remain unchanged for at least 275 d when Asp-Oil is included in the TMR at 25 mg CHBr_3_/kg DMI. The Waygu study demonstrated a tempered but persistent antimethanogenic efficacy compared to the present study and the difference is likely due to the collective effect of lower than optimal inclusion level (25 vs 35 mg CHBr_3_/kg DMI), greater hay inclusion, and a different breed of cattle. This indicates that 17 mg CHBr_3_/kg DMI was below the threshold for persistent antimethanogenic efficacy while 25 mg/kg remained persistent despite a lower initial efficacy. It has been demonstrated that greater proportions of grain compared to grass have a strong influence and improve antimethanogenic efficacy of *Asparagopsis* (Kinley et al., 2021). Furthermore, the emergence of a potential breed effect where large differences exist in feeding systems and genetics may likewise alter the REIL. Consequently, more work may be required to develop breed-specific inclusion levels to achieve target CH_4_ reductions and subsequent emissions accounting for commercial settings. That said, considering studies collectively provides insight on a minimum effective inclusion level (**MEIL**) below which CH_4_ inhibition may be more prone to adaptation. To that end, it was demonstrated in the present study that a lower boundary for Asp-Oil is delivery of approximately 17 mg CHBr_3_/kg DMI. Similarly, the study by Kinley et al. (2020), further explained by Kinley (2018), demonstrated deteriorating antimethanogenic efficacy in the late stages of their study at a slightly lower inclusion level of 13 mg CHBr_3_/kg DMI and was even more so at their lowest level, approaching 7 mg CHBr_3_/kg DMI. However, more research is required to elucidate this phenomenon to confirm the persistence of CH_4_ suppression when approaching the suggested MEIL of approximately 20 mg CHBr_3_/kg DMI. Furthermore, Li et al. (2018) demonstrated persistent CH_4_ mitigation at 0.5% OM kiln-dried *Asparagopsis* (over 72 d; CHBr_3_ delivery not known) and Roque et al. (2021) found no reduction in persistence of CH_4_ mitigation at 0.45% and 0.92% DM FD-Asp (35.1 and 71.5 mg CHBr_3_/kg DMI over 147 d). Collectively, the progression of ruminant feeding studies has emphasized that when feeding *Asparagopsis* products above the lower boundary of the REIL, as demonstrated in the present study, and other dose–response studies (Li et al., 2018; Kinley et al., 2020; and Roque et al., 2021), even for extended periods of over 5 and 9 mo (Roque et al., 2021; Cowley et al., 2023), adaptation leading to loss of antimethanogenic efficacy has not occurred.

Feeding Asp-Oil at the high inclusion level induced a nearly 17-fold increase in dissolved hydrogen as measured in rumen fluid of the heifers and is commensurate with previous findings in an earlier study also demonstrating 98% CH_4_ inhibition using FD-Asp where H_2_ was measured as gas emissions in respiration chambers (Kinley et al., 2020). The former demonstrates the increase in H_2_ pressure in the rumen and the latter demonstrates large quantities of liberated H_2_ are expelled as gas suggesting a balance exists between the two forms. Previously, increased respired H_2_ emissions have been observed with *Asparagopsis* supplementation, but the present experiment is able to demonstrate that without CH_4_ as a sink, H_2_ is also elevated in situ. This demonstrates consistency regarding the fate of H^+^ in CH_4_-inhibited rumens in vivo. In a rumen without CH_4_ inhibition, most H_2_ liberated by fermentative microorganisms is rapidly consumed through interspecies transfer by methanogens in the reduction of CO_2_ into CH_4_ (Wolin et al., 1997). This is reflected in the distinctive increase in H_2_ measured when CH_4_ was effectively eliminated in the present study and that of Kinley et al. (2020). The consumption of H_2_ by methanogens has positive feedback and stimulation of H_2_ liberation from feedstuff thereby perpetuating the interspecies transfer and continuation of CH_4_ production (Wolin et al., 1997). Those authors suggested that interrupting the consumption of H_2_ by methanogens could result in negative influence on acetate production with concomitant impacts on animal growth and performance. Despite the increased dissolved hydrogen recorded in CH_4_-inhibited rumens in this experiment, there was no effect of Asp-Oil on total VFA, acetate and propionate molar concentrations, or acetate:propionate ratio. Yet, past research using FD-Asp has reported variable results relative to whether the diversion of H^+^ from CH_4_ results in an increase in propionate and other VFAs as alternative hydrogen sinks, ranging from no effect to a decrease in acetate:propionate ratio (Ungerfeld, 2015; Li et al., 2018; Kinley et al., 2020; Stefenoni et al., 2021). Despite the accumulation of H_2_, rumen pH and reduction potential were unaffected by the inclusion of Asp-Oil, corresponding with previous work in pellet-fed sheep (Li et al., 2018).

It has been considered that accumulation of H_2_ in the rumen may have deleterious effects on rumen fermentation and consequently G:F (Wolin, 1979; McAllister and Newbold, 2008). This supposition has been challenged by accumulating studies, some already considered here, and as reported in the meta-analysis presented by Ungerfeld (2018) which reported that milk production would increase when adjusted for DMI with 100% inhibition of methanogenesis in dairy cows. To that end, neither this study nor much of the past literature, has demonstrated substantial negative impacts on biological fitness or rumen function in vivo when CH_4_ is inhibited significantly by *Asparagopsis*. Additionally, there have been considerable gains demonstrated in ADG (Kinley et al., 2020) and G:F (Roque et al., 2021) with greater levels of CH_4_ inhibition from feeding *Asparagopsis*, although no changes in either parameter were demonstrated in the present study. The lack of consensus in in vivo findings regarding diversion of H^+^ to beneficial sinks such as propionate, which is an energy precursor in ruminants, indicates a need for more directed research into productive efficiency cobenefits from feeding *Asparagopsis* products in studies that have sufficient animal numbers to confirm relatively small differences (<5%). This study observed no negative effects on cattle when Asp-Oil was included above the MEIL, although, for the avoidance of doubt, this experiment was not designed with sufficient replication or production-relevant management to detect changes in animal performance. Previous studies have reported improved animal performance while offering the caveat that those studies similarly lacked statistical power to detect production responses. There was no effect of Asp-Oil treatment on carcass grading or eating quality, but overall, all animals in this experiment, including control, had relatively small, lean carcasses, relatively low MSA index scores, and consumer eating quality scores unrelated to Asp-Oil. Together with the productivity cobenefits of the antimethanogenic efficacy of Asp-Oil, meat quality impacts need to be resolved in more highly-powered experiments that permit for full expression of productive potential by cattle.

Although an increase in liberated H_2_ due to methanogenesis inhibition by *Asparagopsis* has consistently indicated favorable or neutral response in animal performance, the subsequent loss of H_2_ does represent a loss of feed energy, and conservation of a proportion of that may be further beneficial to G:F. Martinez-Fernandez et al. (2017) demonstrated that the addition of phloroglucinol as an example will stimulate metabolism of H_2_ into acetate and reduce rumen H_2_ pressure, presumably supporting improved G:F. Although the addition of such compounds with *Asparagopsis* is not inherently required, the interaction of such novel compounds with *Asparagopsis* is not known, particularly when methanogenesis is inhibited at levels approaching 100%. Co-supplementation of H_2_ acceptors should be investigated, particularly where CH_4_ inhibition and improving G:F is more challenging, such as in grass-fed systems.

This study demonstrated a considerable margin of safety for the animal consuming Asp-Oil with minimal incidence of change in indicators of animal health or welfare. Most blood cell counts, hemoglobin and haptoglobin concentrations, and fecal glucocorticoid metabolite concentration were unaffected by increasing Asp-Oil inclusion. It has long been considered that CHBr_3_ and presumably other *Asparagopsis* metabolites are antimethanogenic due to reaction with vitamin B_12_ inhibiting the enzyme involved in the methyl transfer of the CH_4_ pathway (Wood et al., 1968). Concomitant depletion of B_12_ and subsequent impact of B_12_ deficiency may be of concern, thus this study quantified B_12_ levels in blood of the Asp-Oil treated heifers. Within the expected considerable variability between individuals in all groups, relative consistency of serum B_12_ was maintained, and there was no difference found compared to the control (Table 5).

Compared to the reference level ranges for bovine hematology reported by Roland et al. (2014) of 1.0 to 6.3 × 10^6^ neutrophils/mL and 160 to 800 × 10^6^ platelets/mL, the High Asp-Oil treatment group was well within the range, and marginally exceeded the range, for plasma neutrophils (4.8 × 10^6^/mL) and platelets (811 × 10^6^/mL), respectively, (Table 5). Due to the widely ranging physiological and environmental characteristics lending to the extensive range, the most appropriate for comparison is a group of untreated animals under similar conditions (Roland et al., 2014). Compared to the control group the Asp-Oil cattle demonstrated a linear increase in both neutrophils and platelets. This may suggest an inflammatory response (Garcia et al., 2017) but remains within the typical range expected. Previous research using FD-Asp found no effect on cellular differentials in sheep (Li et al., 2018). The elevated neutrophils and platelets may be in response to CH_4_ inhibition, elevated dissolved H_2_, or other systemic responses to Asp-Oil and its bioactive compounds. The high concentration of I^-^ present in wild-sourced *Asparagopsis* could potentially interfere with thyroid function and therefore thermoregulation (Arthur and Beckett, 1999), but T3 and T4 concentrations, and parameters of temperature circadian rhythm were unaffected by Asp-Oil inclusion.

Present in some heifers of all treatment groups including control, were incidences of inflammation, hyperkeratosis of rumen papillae, and variable physiological changes to rumen mucosa as is consistent with feeding high-grain diets (Magrin et al., 2021). Two occurrences of rumen scaring associated with unidentified rumen anomaly predating the study were observed. Subacute rumen acidosis (**SARA**) is a common attribute of feeding high levels of grains with low levels of fiber (Herrero et al., 2014) and the scope of associated rumen hyperkeratosis and ruminitis observed in 2,161 rumens from grain-fed cattle inspected by Magrin et al. (2021) was 58% and 30%, respectively, and incidence of scaring and ulceration were evident and less prevalent at 15% and 0.4%, respectively, (Magrin et al., 2021). Therefore, SARA-exacerbated rumen afflictions are hallmarks of feedlot feeding systems. Considering that some rumen abnormalities occurred among animals of all groups, it is not possible to attribute cause and effect relationship between the use of Asp-Oil and the ruminal lesions in the present study. That said, the commonality and manifestation of ruminal parakeratosis in this study was typically mild in all groups and the High Asp-Oil group had no incidences of considerable change and only one incidence of more severe manifestation occurred in the medium Asp-Oil group. Furthermore, Muizelaar et al. (2021) supplemented dairy cows with FD-Asp mixed with wheat and beet pulp without offering any other feed for up to 2 h and described significant ruminal abnormalities and that they recommended may not be related to the supplementation of FD-Asp. The researchers had responded to the cow’s suppressed intake of the full offering of their highly concentrated FD-Asp mix with augmented feed restriction to necessitate intake of the supplement. This may have contributed to increased probability of SARA manifestation and their subsequent assertion that the abnormalities may not be related directly to FD-Asp. Further studies of Asp-based supplements may continue to perform ruminal wall histology to monitor the effects of different supplements. Our results also highlight the importance of a control group with the same feedlot regime, minus the Asp-based supplement. When ruminal abnormalities are present even in the control group, then strategies to reduce SARA and improve animal welfare may be recommended industry-wide, regardless of Asp-based supplementation.

There was no evidence of CHBr_3_ transfer to meat or feces in any Asp-Oil treatment in this study, supporting previous research demonstrating that *Asparagopsis*-derived CHBr_3_ does not transfer to meat and edible offal (Li et al., 2018; Kinley et al., 2020), feces or milk from healthy animals (Muizelaar et al., 2021) when offered at effective feed inclusion levels. Inclusion rates of Br^−^ in all diets were well below the threshold of maximum tolerable level (**MTL**) concentration (NASEM, 2016). The increasing dietary Br^−^ concentration observed with increasing Asp-Oil treatment was reflected in serum Br^−^concentrations. Bromine is rapidly excreted through the urine, and there was no evidence of upregulated transfer of Br^−^ to feces with Asp-Oil treatment in the present study. In the present study, Br^−^ residues in the carcass increased with increasing Asp-Oil treatment in the kidney, liver, and striploin. Fat was not a point of deposition of Br^−^ based on the results of the present study. Even the greatest carcass Br^−^ concentration observed (kidney, High Asp-Oil treatment) is unlikely to result in Br^-^ intakes exceeding the recommended upper limit: excessive levels of Br^−^ intake would require daily consumption of 0.42 kg/d (< 3 yr old) to 2.3 kg/d (19 + yr old) of kidney from that carcass (FAO, 1999). Further research is recommended to elucidate if a preslaughter withhold (2 to 3 d) of *Asparagopsis* would further lower the level of Br^−^ in meat and edible offal.

Finishing cattle have an I^−^ requirement of 0.50 mg/kg DM, and 50 mg/kg DM has been suggested as the MTL for beef cattle (NASEM, 2016). The accumulation of I^-^ in seaweeds can potentially be quite high and in some circumstances when fed at high levels may exceed the MTL (Kinley et al., 2020). That said, all diets in the present experiment contained considerably less I^−^ than the recommended MTL at < 10 mg I/kg DM. Serum I^−^ was reduced with high Asp-Oil inclusion, but this is likely a type I error, as no effect of Asp-Oil treatment on thyroid function was observed (National Research Council, 1980). Increasing Asp-Oil treatment resulted in increased I^−^ concentration in feces, such that the feces of High Asp-Oil heifers was 77% greater than control heifers; however, >90% of ingested iodine that is not concentrated in the thyroid is excreted via urine (Institute of Medicine Panel on Micronutrients, 2001). In the carcass, I^−^ concentrations were below detectable limits in most samples of liver, and all samples of fat and striploin. The sampling site of greatest concentration of I^−^ was the kidney in all treatments, although in most samples, based on expected consumer intake of these products this was below the recommended sustained upper limit (mg/d) of iodine intake (Trumbo et al., 2001).

## Conclusion

As the first test of Asp-Oil in beef cattle was equally efficacious at inhibiting enteric CH_4_ production as previous studies using FD-Asp, on a CHBr_3_ mg/kg DMI basis, while also being safe for animals and consumers of beef. This study demonstrated the functional REIL and MEIL to achieve complete suppression of CH_4_ production in cattle fed a feedlot diet containing Monensin and oil was approximately 34 mg CHBr_3_/kg DM, however further testing of inclusion levels between 17 and 34 mg CHBr_3_/kg DM may refine this estimate. Increasing inclusion of Asp-Oil did not result in differences in animal health, production, carcass parameters, or meat-eating quality, and testing for CHBr_3_, I^−^, and Br^−^ residues found no risks to consumers of meat or offal. Suppression of CH_4_ production resulted in accumulation of dissolved hydrogen in the rumen fluid, without changes to acetate or propionate synthesis, or effect on ADG or G:F.

## Supplementary data

Supplementary data are available at *Journal of Animal Science* online.

skae109_suppl_Supplementary_Materials

## Abbreviations

ADG: average daily gain
Asp-Oil: *Asparagopsis taxiformis* bioactives stabilized in canola oil
BW: body weight
CH_4_: methane
CHBr_3_: , bromoform
CO_2_: carbon dioxide
DM: dry matter
DMI: dry matter intake
FD-Asp: freeze-dried *Asparagopsis*
G:F: gain:feed
H_2_: hydrogen gas
MEIL: minimum effective inclusion level
MESOR: midline statistic of rhythm
MSA: meat standards Australia
MTL: maximum tolerable level
pH_u_: ultimate pH
REIL: range of effective inclusion levels
SARA: subacute ruminal acidosis
TMR: total mixed ration
