# Supplementary material for: Bioactive metabolites of Asparagopsis stabilized in canola oil completely suppress methane emissions in beef cattle fed a feedlot diet
Source: J Anim Sci. 2024 Apr 22;102:skae109. doi: 10.1093/jas/skae109 (PMC11088279; doi:10.1093/jas/skae109)
Supplement: skae109_suppl_Supplementary_Materials [file skae109_suppl_supplementary_materials.docx]

Supplementary material

Table S1. Formulated bromoform (CHBr_3_) inclusion (mg/kg DM) in during adaptation period.

|  | Treatment | | | |
| --- | --- | --- | --- | --- |
| Experiment days | Control | Low Asp-Oil | Medium Asp-Oil | High Asp-Oil |
| 1-7 (Starter) | 0 | 4.2 | 8.5 | 12.7 |
| 8-15 (Intermediate I) | 0 | 8.5 | 17.0 | 25.5 |
| 16-22 (Intermediate II) | 0 | 12.7 | 25.5 | 38.2 |

Table S2. Daily methane production (g CH_4_/day) of Angus heifers (least squares mean ± s.e.) fed increasing levels of Asparagopsis bioactives stabilized in a canola oil carrier (Asp-Oil) in each diet during the adaptation period.

|  | Asp-Oil Treatment^1^ | | | |  | | *P*-value^2^ | | |
| --- | --- | --- | --- | --- | --- | --- | --- | --- | --- |
| Diet | Control | Low | L | Q | |  | Linear | Quadratic |  |
| Starter | 62.20 ± 14.43 | 50.90 ± 13.24 | 55.60 ± 13.24 | 18.90 ± 16.20 | |  | 0.263 | 1.000 |  |
| Intermediate I | 53.80 ± 12.06 | 21.51 ± 11.07 | 0.12 ± 11.07 | 0.62 ± 13.54 | |  | 0.022 | 0.463 |  |
| Intermediate II | 90.75 ± 13.62 | 15.11 ± 12.84 | 0.00^*^ ± 12.84 | 3.62 ± 14.09 | |  | <0.001 | 0.203 |  |

^1^Formulated CHBr_3_ inclusions in diet DM were (for Starter, Intermediate I, Intermediate II and Finisher, respectively): Control = 0 mg CHBr_3_/kg DM, n = 4; Low = 4.2, 8.5. 12.7 and 16.9 mg CHBr_3_/kg DM; n = 5; Medium = 8.5, 17.0, 25.5, and 34.0 mg CHBr_3_/kg DM, n = 5; High = 12.7, 25.5, 38.2, and 51.0 mg CHBr_3_/kg DM, n = 4; ^2^Bonferroni-Holm-adjusted. CH_4_ measured on day 6 of each diet of the adaptation period. *For the intermediate II medium treatment the statistical model least-squared means were predicted at -1.45 ± 12.84, but is presented as 0.00 ± 12.84.

Table S3. Methane yield (g CH_4_ /kg dietary dry matter intake) of Angus heifers (least squares mean ± s.e.) fed increasing levels of Asparagopsis bioactives stabilized in a canola oil carrier (Asp-Oil) in each diet during the adaptation period.

|  | Asp-Oil Treatment^1^ | | | |  | *P*-value^2^ | |
| --- | --- | --- | --- | --- | --- | --- | --- |
| Diet | Control | Low | Medium | High |  | Linear | Quadratic |
| Starter | 9.95 ± 2.46 | 10.50 ± 2.25 | 9.10 ± 2.25 | 2.92 ± 2.75 |  | 0.213 | 0.400 |
| Intermediate I | 7.75 ± 1.94 | 3.82 ± 1.77 | 0.02 ± 1.77 | 0.12 ± 2.17 |  | 0.036 | 0.728 |
| Intermediate II | 10.42 ± 1.53 | 2.16 ± 1.44 | 0.00^*^ ± 1.44 | 0.39 ± 1.58 |  | <0.001 | 0.251 |

^1^Formulated CHBr_3_ inclusions in diet DM were (for Starter, Intermediate I, Intermediate II and Finisher, respectively): Control = 0 mg CHBr_3_/kg DM, n = 4; Low = 4.2, 8.5. 12.7 and 16.9 mg CHBr_3_/kg DM; n = 5; Medium = 8.5, 17.0, 25.5, and 34.0 mg CHBr_3_/kg DM, n = 5; High = 12.7, 25.5, 38.2, and 51.0 mg CHBr_3_/kg DM, n = 4;  ^2^Bonferroni-Holm-adjusted. CH_4_ measured on day 6 of each diet of the adaptation period. *For the intermediate II medium treatment the statistical model least-squared means were predicted at -0.16 ± 1.44, but is presented as 0.00 ± 1.44.

Table S4. Complete data of heifers removed from analysis of the experimental period. Heifer #19 (Control) showed signs of sub-acute acidosis and was removed from the experiment on d 46. Heifer #76 (High Asp-Oil) completed the experiment, but due to persistent low intake and other indications of sub-acute acidosis, she was excluded from analysis of the experiment.

| Animal ID | 19 (Control) | 76 (High Asp-Oil) |
| --- | --- | --- |
| Methane (CH_4_) emissions, mean |  |  |
| Total CH_4_, g/d | 76.55 | 5.59 |
| CH_4_ yield, g/kg DMI | 12.88 | 2.57 |
| Animal performance |  |  |
| DMI, kg/d | 6.27 | 3.51 |
| Initial LW, kg | 324 | 356 |
| Final LW (d 80/81), kg | 366 | 342 |
| Average daily gain (kg/day) | -3.4 | -2.0 |
| Rumen function measures, mean |  |  |
| pH | 5.94 | 5.54 |
| Reduction potential | -33 | -110 |
| Faecal starch (%) | 3.6 | 3.3 |
| Protozoa counts, mean |  |  |
| Total protozoa (×10^3^/mL) | 4.0 | 2.3 |
| Large holotrich (×10^3^/mL) | 0.0 | 2.0 |
| Small holotrich (×10^3^/mL) | 0.0 | 0.0 |
| Entodiniomorphs (×10^3^/mL) | 4.0 | 0.3 |
| Rumen volatile fatty acid molar proportions, mean |  |  |
| Total VFAs (mmol/L) | 74.2 | 53.5 |
| Acetic acid (mmol/mmol) | 0.52 | 0.43 |
| Propionic acid (mmol/mmol) | 0.19 | 0.35 |
| Butyric acid (mmol/mmol)) | 0.25 | 0.17 |
| Iso-Butyric acid (µmol/mmol) | 0.01 | 0.01 |
| Valeric acid (µmol/mmol) | 0.02 | 0.01 |
| Iso-Valeric acid (µmol/mmol) | 0.01 | 0.03 |
| Caproic acid (µmol/mol) | 0.02 | 0.00 |
| Acetate:Propionate | 2.82 | 1.33 |
| Rumen ammonium-N (mg/L, mean) | 22.9 | 323.9 |
| Blood cell concentrations of plasma, mean |  |  |
| Leucocytes (×10^6^/mL) | 7.9 | 12.6 |
| Neutrophils (×10^6^/mL) | 2.2 | 5.1 |
| Lymphocytes (×10^6^/mL) | 7.8 | 6.0 |
| Monocytes (×10^6^/mL) | 0.74 | 0.85 |
| Eosinophils (×10^6^/mL) | 0.11 | 0.49 |
| Basophils (×10^6^/mL) | 0.09 | 0.15 |
| Erythrocyte (×10^9^/mL) | 7.2 | 8.4 |
| Haemoglobin g/dL | 11.5 | 12.3 |
| Haptoglobin (mg/L) | 415.5 | 305.1 |
| Haemaocrit (%) | 34.2 | 35.4 |
| Mean corpuscular volume (fL) | 42.0 | 42.0 |
| Mean corpuscular haemoglobin (pg) | 14.1 | 14.4 |
| Mean corpuscular hemoglobin concentration (g/dL) | 33.5 | 34.2 |
| Platelets (×10^6^/mL) | 493.5 | 690.7 |
| Hormone and vitamin B12 metabolism |  |  |
| Thyroxine, ng/mL | 67.7 | 70.3 |
| Triiothyronine, ng/mL | 2.1 | 1.3 |
| Faecal cortisol, ng/g | 1.15 | 0.90 |
| Serum Vitamin B_12_ , *p*g/mL | 33.69 | 59.93 |
| Serum residues, mg/L, mean |  |  |
| Iodine | 1.29 | 0.71 |
| Bromide | 8.38 | 29.04 |
| Faecal residues, mg/kg, mean |  |  |
| Iodine | 0.42 | 0.71 |
| Bromide | 228.7 | 269.7 |
| Bromoform (CHBr_3_) concentration (mg/kg) |  |  |
| Kidney | ND | ND |
| Liver | ND | ND |
| Fat | ND | ND |
| Striploin | ND | ND |
| Bromide (Br^-^) concentration^1^ (mg/kg) |  |  |
| Kidney | 13 | 14 |
| Liver | 6 | 8 |
| Fat | ND | ND |
| Striploin | ND | ND |
| Iodide (I^-^) concentration^1^ (mg/kg) |  |  |
| Kidney | 0.1 | 0.1 |
| Liver | ND | ND |
| Fat | ND | ND |
| Striploin | ND | ND |
| Rumen wall morphology |  |  |
| Papillae colour  (1-5 scale) | 0.5* | 0 |
| Papillae shape (1-4 scale) | 1* | 0 |
| Ventral sac damage (1-5 scale) | 0* | 0 |
| Carcase grading |  |  |
| HSCW, kg | 176.4* | 151.6 |
| Dressing percentage, % | 48.1* | 44.7 |
| P8 fat, mm | 6* | 3 |
| Rib fat, mm | 4* | 2 |
| EMA, cm2 | 43* | 74 |
| MSA Marbling score | 400* | 230 |
| AUSMEAT Marbling score | 2* | 0 |
| AUSMEAT fat colour score | 1* | 1 |
| AUSMEAT meat colour score | 5* | 6 |
| Ossification score | 140* | 140 |
| pH_u_ | 5.65* | 5.76 |
| MSA index | 56.2* | - |
| Shear force, kg | 4.66* | 7.42 |

ND: Below limits of detection; *Heifer #19 was removed from the experiment on d 46 and thereafter maintained on a roughage-based diet, and was slaughtered with the remaining experimental heifers.
